# Applications of System Dynamics Models in Chronic Disease Prevention: A Systematic Review

**DOI:** 10.5888/pcd18.210175

**Published:** 2021-12-23

**Authors:** Ying Wang, Bo Hu, Yuxue Zhao, Guofang Kuang, Yaling Zhao, Qingwei Liu, Xiuli Zhu

**Affiliations:** 1 School of Nursing, Medical College, Qingdao University, Qingdao, China; 2 Department of Thoracic Surgery, Qingdao Municipal Hospital, Qingdao, China; 3 Department of Obstetrics and Gynecology, Affiliated Hospital of Qingdao University, Qingdao, China

## Abstract

**Introduction:**

Chronic disease is a serious health problem worldwide. Given that health care resources are limited, a comprehensive, effective, and affordable way is needed to provide insights to prevent chronic diseases. System dynamics models provide a comprehensive and systematic method that can predict results over time. These models can simulate and predict appropriate prevention measures for chronic diseases to determine the best practice.

**Methods:**

Two researchers (Y.W., B.H.) independently searched databases (PubMed, Web of Science, Scopus, and Embase) for full-text articles published from January 2000 through February 2021. A PRISMA (Preferred Reporting Items for Systematic Reviews and Meta-Analysis) 2020–compliant search was carried out to review system dynamics models of chronic disease prevention. A total of 34 articles were included in our study.

**Results:**

We divided the prevention measures of system dynamics models into 2 main categories: upstream prevention and downstream prevention. Upstream prevention measures include lifestyle (eg, tobacco control, balanced diet, mental health, moderate exercise), obesity prevention, and social factors. Downstream prevention measures include clinical treatment of chronic diseases. Results showed that effective upstream prevention measures could reduce the prevalence of chronic diseases, and downstream prevention measures could reduce the incidence of complications, improve quality of life, prolong life, save medical costs, and reduce mortality.

**Conclusion:**

To our knowledge, our systematic review is the first to evaluate the application of system dynamics models in preventing chronic diseases. Such models can provide effective simulations. Hence, we can use system dynamics models to design and implement effective prevention measures for people with chronic diseases.

**Summary**

**What is already known about this topic?**

The prevention of chronic diseases is one of the most critical health problems in the world.

**What is added by this report?**

Our study identified the potential short- and long-term effects of upstream and downstream chronic disease prevention strategies through a systematic review.

**What are the implications for public health practice?**

Health care workers and policy makers can use system dynamics models to analyze the priorities of chronic disease prevention to delay disease progression and reduce the health care burden of chronic diseases.

## Introduction

Chronic diseases have the highest disease mortality worldwide, and their prevention is affected by many driving forces, such as lifestyle, health care, and health policies (1). System dynamics models can help us understand the complex relationships between prevention measures and chronic diseases. System dynamics modeling is a system simulation method that describes the structure and dynamics of complex systems (2). Systems are interconnected to produce their own pattern of behavior over time and focus on the whole problem, its structure, and its dynamics rather than its parts and its static state. The prevention of chronic diseases involves many variables and stakeholders. These variables form a highly complex system with complex dynamic changes and interactions. When we implement a seemingly ideal solution to a problem, that solution often results in failure or more serious consequences, because dynamic complexity leads to policy resistance (2,3). Policy resistance means that implementing prevention measures can produce outcomes opposite to those expected. For example, antibiotics can cure bacterial infections, but antibiotic abuse stimulates the production of drug-resistant bacteria (2). That is what Sterman (2) meant when he said, “today’s interventions have become tomorrow’s problems.” In general, the behavior of complex systems is often counterintuitive, which means it can lead to unexpected consequences (2–4).

System dynamics models can help us establish a holistic concept and analyze the prevention of chronic diseases as a whole. The steps for establishing system dynamics models are 1) problem definition, 2) development of a conceptual model, 3) development of a quantitative model, 4) validation and testing, and 5) policy simulation. In the process of developing the model, stakeholders continue to gather qualitative and quantitative evidence to optimize the model. Homer and Hirsch (4) propose that the prevention of chronic diseases mainly focuses on 2 parts: upstream prevention and downstream prevention. Upstream prevention focuses on preventing the occurrence of diseases, and its focus is on people without chronic diseases. Downstream prevention refers to the prevention of complications of chronic diseases, and focuses on people with chronic diseases. Therefore, our study discussed and analyzed upstream and downstream prevention according to chronic disease prevention. Our objective was to systematically evaluate and describe the potential short- and long-term effects by using system dynamics models to model the upstream and downstream prevention of chronic diseases. We theorized that examined evidence could provide health care workers with prevention measures for people with chronic diseases.

## Methods

**Figure 1:**
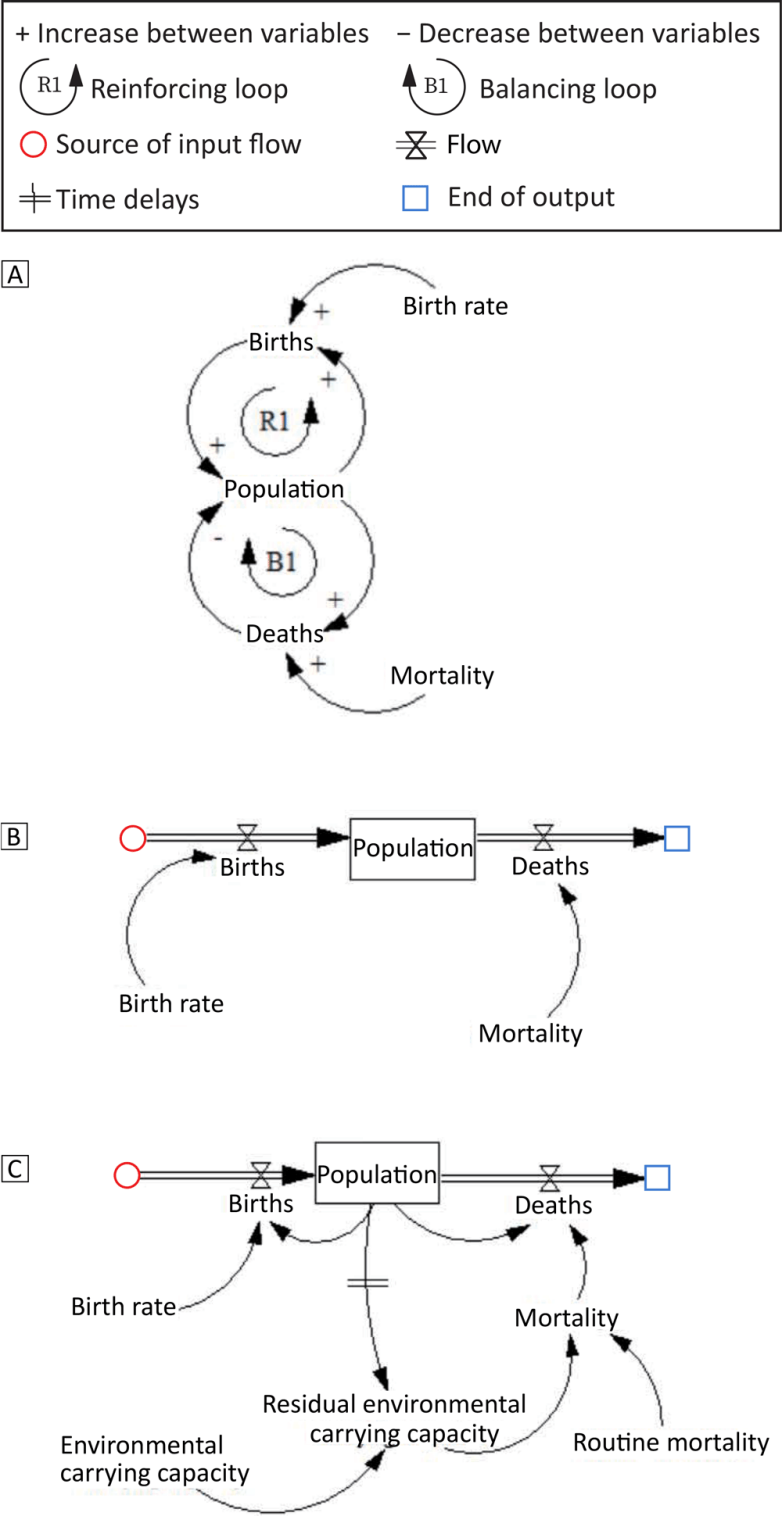
System dynamics model in 3 parts showing the convergence of births and deaths to create population. The variables are linked by a causal chain with positive (+) and negative (–) polarity. The positive sign indicates that when variable A increases, variable B also increases; the negative sign indicates that when variable A increases, variable B decreases. The positive and negative signs represent either increase or decrease, not the proportional relationship between variables. Part A is a causal loop diagram that shows a reinforcing loop for increases in births and a balancing loop for deaths. Part B is a stock-flow diagram illustrating the convergence of birth rate and mortality rate, which equals population. Part C is a hybrid diagram that incorporates the effect of environmental carrying capacity, residual environmental carrying capacity, and routine mortality on births and deaths to result in population.

We developed a causal loop diagram, a stock-flow diagram, and a hybrid diagram by using Vensim PLE software (Ventana Systems, Inc) to provide examples of common system dynamics modeling conventions (Figure 1). The causal loop diagram is the first stage of the conceptual model and is a dynamic feedback process (5). After a comprehensive analysis of the identified problems, stakeholders establish a causal loop diagram to qualitatively show the causal relationship between variables. In our example, population and births form a reinforcing feedback (loop R1, Figure 1A). Changes generated by the population will affect births and feedback to the population. Similarly, the population and deaths form a balancing feedback, loop B1. The causal loop diagram is widely used in the initial stage of modeling, but it is not necessary for experienced modelers. The stock-flow diagram (Figure 1B) is developed from the causal loop diagram and describes stock variables (cumulative, indicating system status), flow variables (indicating stock changes), auxiliary variables (to help express other information), and constant variables (constant values). We first determine the main relationship between stock variables (population), flow variables (birth and death), auxiliary variables, and constant variables (birth rate, mortality rate). Then, the lookup function is used to determine the nonlinear relationship between variables, and the parameters of various variables are estimated and assigned. The hybrid diagram (Figure 1C) combines the causal loop diagram with the stock-flow diagram, which not only expresses the important stock and flow variables, but also maintains the simplicity of the causal loop diagram.

Time delays are an important concept in system dynamics models, which means that the prevention measures we implement will not have an immediate effect. For example, time delays can occur between population and residual environmental carrying capacity (Figure 1C). Time delays in the feedback loop will cause system instability, lead to overshoot or oscillation, and reduce our ability to learn and accumulate experience (2,3).

### Data sources

We conducted our systematic review in accordance with the guidelines of PRISMA (Preferred Reporting Items for Systematic Reviews and Meta-Analysis) 2020 (6). PubMed, Scopus, Embase, and Web of Science databases, from 2000 to the present, were searched initially in January 2020 and searched again in February 2021 for potentially relevant research published in English. We conducted the search using medical subject headings (MeSH terms) and free-text words. The search strategy was “noncommunicable diseases” OR “chronic disease” OR “chronic illness” OR “chronic disease [MeSH Terms]” OR “noncommunicable diseases [MeSH Terms]” AND “system dynamics” OR “computer simulation [MeSH Terms]” OR “dynamics, nonlinear [MeSH Terms].”

### Study selection

The inclusion criteria for our study were 1) original studies or study protocols published in the database searched, 2) studies reporting chronic disease prevention based on system dynamics models, 3) studies including human participants, and 4) studies published in English. The exclusion criteria were 1) abstracts and conference proceedings and 2) studies investigating nonchronic diseases, such as emergency care, epidemic prediction, and vaccination.

**Figure 2:**
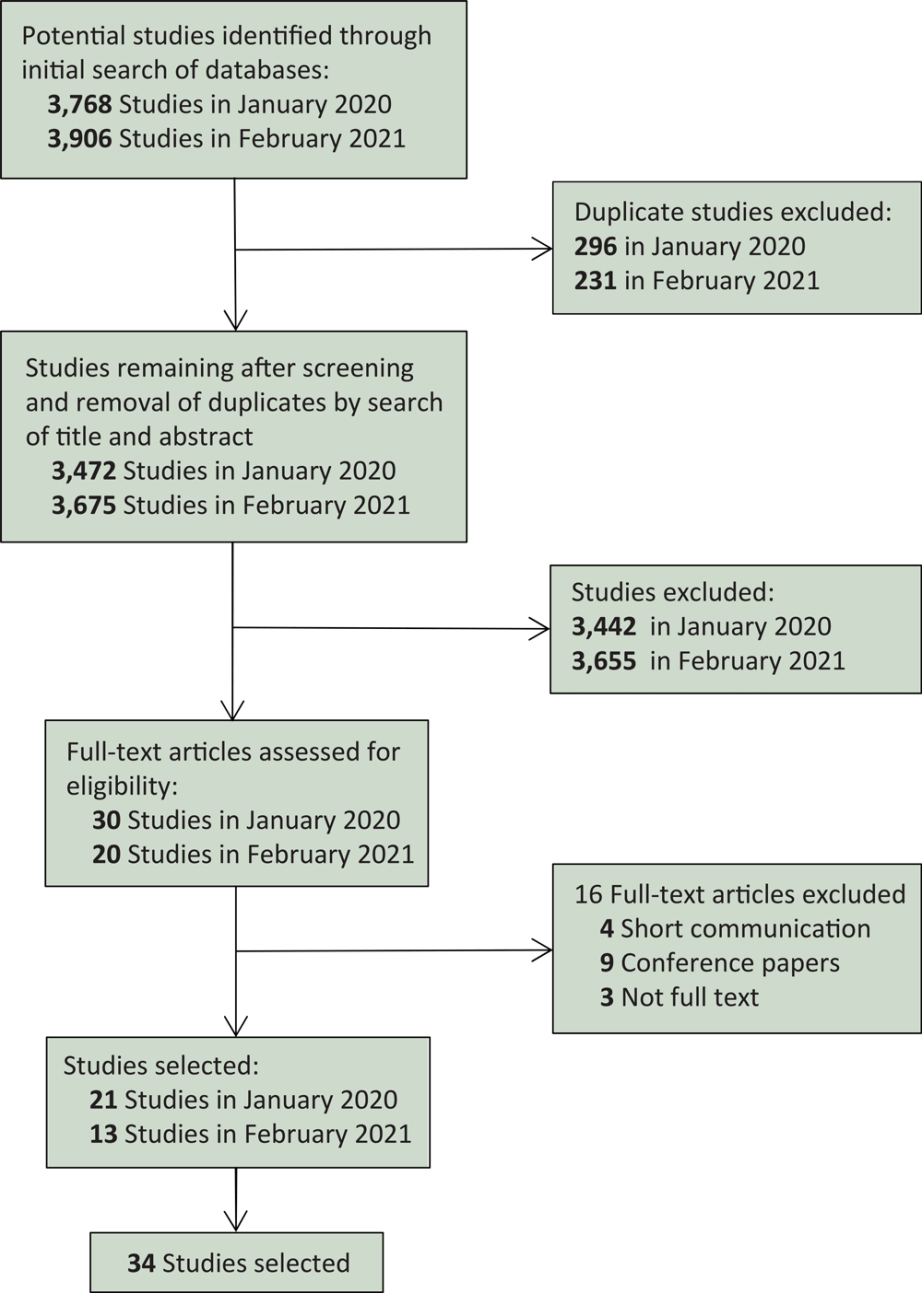
Selection process for study of system dynamics models in chronic disease prevention, January 2000 to February 2021. Preferred Reporting Items for Systematic Reviews and Meta-Analyses (PRISMA) diagram showing research study identification and selection process.

Two researchers (Y.W., B.H.) formulated a comprehensive search strategy to conduct the literature search. Duplicates were independently removed by using EndNote reference manager (Clarivate), and abstracts and full texts were reviewed to remove ineligible studies. Finally, the identified studies were retrieved and aggregated for review during the preliminary search in January 2020 and the repeated search in February 2021 (Figure 2).

### Data extraction

We developed a data form to collect the following information: study authors and year of study, country where the research was conducted, study objective, type of upstream prevention, and type of downstream prevention (Table 1). Two researchers independently extracted data from the selected articles. Differing opinions were resolved though discussion. In addition, we used the assessment criteria from a previous study (7) to evaluate the quality of the literature. Our 8 quality criteria were 1) presenting a clear objective; 2) presenting clear scenarios and interventions; 3) presenting clear outcome variables by graphs, charts, or tables; 4) describing the development of a system dynamics model framework or presenting a detailed model framework; 5) presenting and explaining model parameters; 6) improving the quality of data by using stakeholders’ engagement, surveys, interviews, and databases; 7) validating models (validation used 4 methods: sensitivity testing parameters that the model is highly sensitive to and comparing them with the real world; model data calibration to compare model data with the real world; a structural test to compare mathematical formulas or logical relationships in the model with the real world; and a behavior pattern test to evaluate the accuracy of model prediction to exchange results and achieve goals (8)); and 8) presenting clear results.

**Table 1:** Summary of Articles Reviewed, Systematic Review of Applications of System Dynamics Models in Chronic Disease Prevention, January 2000–February 2021

Author and Year	Country	Objective	Predictors	
			Upstream Prevention^a^	Downstream Prevention^b^
Homer et al, 2006 (4)	US	Explore the relationship between sick population and resource utilization	Upstream prevention of disease	Downstream clinical treatment
Guariguata et al, 2016 (9)	Caribbean Community	Develop a system dynamics model to guide diabetes prevention and control policies	Obesity management Physical inactivity Diet	—
Ansah et al, 2019 (10)	Singapore	Explore and reach a consensus on the management of chronic diseases	Population ageing Tobacco use Unhealthy diets Physical inactivity	Medicine Cost of treatment Access to clinics Quality of care
Allender et al, 2015 (11)	Australia	Establish a causal loop diagram of factors affecting children's obesity in the community through the group model building	Social influences Fast food Junk food Physical activity	—
Homer et al, 2008 (12)	US	Develop a system dynamics model to predict the risk factors of CVD	Primary care Healthy food option Physical activity Smoking regulation Reduce chronic stress	—
Ansah et al, 2018 (13)	Singapore	Describe the dynamics complexity of chronic disease care	Primary health care	Clinical care Outpatient care
Lounsbury et al, 2014 (14)	US	To explain the effectiveness of system dynamics in promoting the quality of life of chronic disease	Qualitative research	—
Kang et al, 2016 (15)	US	To investigate how systematic thinking supports nursing intervention decision-making in the management of CKD	—	Physician education Care coordination Care manager education
Sugiyama et al, 2017 (16)	Japan	Predict the number of people with diabetes and the number of people who need dialysis because of diabetic nephropathy	Diabetes prevention and management	End-stage renal disease prevention
Kang et al, 2017 (17)	US	To study the influence of system dynamics method on nursing intervention of CKD patients	—	Physician education Care coordination Care manager education
Vanderby et al, 2015 (18)	Canada	Simulation of complete continuous care	—	Continuous care
Kuo et al, 2016 (19)	US	The Prevention Impacts Simulation Model (PRISM) was used to simulate population health outcomes	Healthy eating Active living	—
Honeycutt et al, 2019 (20)	US	Estimate the potential impact of Communities Putting Prevention to Work tobacco intervention on avoiding deaths and medical costs by 2020	Tobacco control Secondhand smoke exposure	—
Soler et al, 2016 (21)	US	Analyze the short-term and potential long-term benefits of Communities Putting Prevention to Work	Obesity and tobacco use Secondhand smoke exposure	—
Fallah-Fini et al, 2014 (22)	US	Using system dynamics model to quantify the energy imbalance gap leading to obesity in American adults	Energy estimate	—
Honeycutt et al, 2015 (23)	US	Reported on results of the strategy to reduce the impact of chronic diseases on communities	Behavioral support Taxes and regulation Health promotion and access	Clinical
Homer et al, 2014 (24)	US	Compare the potential of emerging interventions and existing interventions to reduce cardiovascular risk factors	Air Lifestyle Care	Air: post-CVD Lifestyle: post-CVD Care: post-CVD
Homer et al, 2010 (25)	US	Used the system dynamics model to evaluate risk factors for the management of CVD	Care/air/lifestyle Weight-loss and mental health services	—
Hirsch et al, 2010 (26)	US	The factors leading to cardiovascular events for the first time were simulated and modeled	Lifestyle and environmental Mental and medical health care	Mental and medical health care: post-CVD
Hirsch et al, 2014 (27)	US	The Prevention Impacts Simulation Model (PRISM) predicts the different consequences of interventions to reduce the risk of cardiovascular disease	Behavioral support Health promotion and access Tobacco taxes and regulation	Clinical: post-CVD Behavioral support: post-CVD
Loyo et al, 2013 (28)	US	Coordination of community prevention efforts using a system dynamics model for CVD risk	Air (tobacco control air pollution reduction) Comprehensive nursing Improve lifestyle	—
Yarnoff et al, 2019 (29)	US	Investigate the long-term effect of clinical and community intervention	Community intervention	Clinical intervention
Chen et al, 2018 (30)	US	Simulate and predict the potential impact of socio-economic programs on obesity rates	Employment rate Family income level	—
Brittin et al, 2015 (31)	US	Simulate the potential of social factors to prevent chronic disease in low-income urban communities	Social factors such as income and employment, neighborhood attraction, and social cohesion	Manage cases of chronic disease effectively
Apostolopoulos et al, 2018 (32)	US	Explore the factors that affect the health of Black Americans	Unemployment Limited access to health care Socioeconomic inequality	—
Milstein et al, 2007 (33)	US	Explain the trend of diabetes prevalence in US and predict the trend before 2010	Glycemic screening Reduce obesity rate Prediabetes management	Diabetes management
Jones et al, 2006 (34)	US	Explain the growth of diabetes and predict future growth	Reduce the rate of obesity	Enhance clinical management of diabetes and prediabetes
Ansah et al, 2019 (35)	Singapore	Evaluate the effects of hypertension and diabetes management and smoking cessation intervention on cardiovascular event	Diabetes management Hypertension management Smoking cessation	—
Cruz et al, 2019 (36)	Colombia	Used the causal loop diagram to analyze the kidney procurement system in Colombia	—	Kidney donation
Homer et al, 2004 (37)	US	Used the system dynamics model to simulate the cost-effective results of diabetes and heart failure	Screening and prevention education for diabetes	Disease clinical care Risk management for heart failure
Diaz et al, 2015 (38)	US	A simulated intervention study on triage of patients with chronic diseases from the emergency department	—	Insurance coverage Visit rate
Mishra et al, 2018 (39)	India	Using system dynamics model to predict the increase of prevalence rate of diabetes mellitus	Upstream prevention	Downstream treatment
Homer et al, 2007 (40)	US	To explain the rising prevalence of chronic disease and responses to it	Upstream prevention	Downstream care
Diaz et al, 2015 (41)	US	Simulation of the cost saving of intervention in a well-defined population	—	Chronic disease management cost-effectiveness

Abbreviations: —, not applicable; CKD, chronic kidney disease; CVD, cardiovascular disease.

a Upstream prevention measures are lifestyle (eg, tobacco control, balanced diet, mental health, moderate exercise), obesity prevention, and social factors.

b Downstream prevention measures are clinical treatment and care of chronic diseases.

Because of the differences in evaluation indicators involved in qualitative and quantitative models, we used only 5 of our 8 quality criteria (criteria 1, 4, 5, 6, and 8) to assess the quality of qualitative research. We used all 8 criteria to evaluate quantitative research. The score for each item ranged from 0 to 2 (0 = not mentioned, 1 = mentioned, and 2 = fully described). Therefore, the total scores for qualitative and quantitative research were 10 and 16 points, respectively. To further assess the quality, we converted the research quality score into a percentage. The percentage was the study scores divided by the total point score for that category (10 for qualitative, 16 for quantitative) and multiplied by 100% (Table 2). The quality assessment criteria of the reviewed studies were as follows: good quality, >80%; medium quality, 70%–80%; poor quality, 65%–70%; and very poor quality, <65% (7). We omitted articles of poor and very poor quality.

**Table 2:** Quality Assessment of Reviewed Articles, Systematic Review of Applications of System Dynamics Models in Chronic Disease Prevention, January 2000–February 2021^a^

Author, Year, Type^b^	Quality Criteria Score^c^								Score	Study Score^d^, %
	1	2	3	4	5	6	7	8		
Homer et al, 2006, quantitative (4)	2	2	2	2	1	0	0	2	11	68.8
Guariguata et al, 2016, qualitative (9)	2	—	—	2	1	1	—	2	8	80.0
Ansah et al, 2019, qualitative (10)	2	—	—	2	2	1	—	2	9	90.0
Allender et al, 2015, qualitative (11)	2	—	—	2	2	1	—	2	9	90.0
Homer et al, 2008, qualitative (12)	2	—	—	2	1	1	—	2	8	80.0
Ansah et al, 2018, qualitative (13)	2	—	—	2	2	1	—	2	9	90.0
Lounsbury et al, 2014, qualitative (14)	2	—	—	2	2	1	—	2	9	90.0
Kang et al, 2016, qualitative (15)	2	—	—	2	2	1	—	2	9	90.0
Sugiyama et al, 2017, quantitative (16)	2	2	2	2	2	1	2	2	15	93.8
Kang et al, 2017, quantitative (17)	2	2	2	2	2	1	1	2	14	87.5
Vanderby et al, 2015, quantitative (18)	2	2	2	2	2	2	2	2	16	100.0
Kuo et al, 2016, quantitative (19)	2	2	2	1	2	2	2	2	15	93.9
Honeycutt et al, 2019, quantitative (20)	2	2	2	1	2	1	2	2	14	87.5
Soler et al, 2016, quantitative (21)	2	2	2	1	1	1	2	2	13	81.3
Fallah-Fini et al, 2014, quantitative (22)	2	2	2	2	2	1	2	2	15	93.8
Honeycutt et al, 2015, quantitative (23)	2	2	2	1	2	1	2	2	14	87.5
Homer et al, 2014, quantitative (24)	2	2	2	1	2	2	2	2	15	93.8
Homer et al, 2010, quantitative (25)	2	2	2	2	2	2	2	2	16	100.0
Hirsch et al, 2010, quantitative (26)	2	2	2	2	2	1	1	2	14	87.5
Hirsch et al, 2014, quantitative (27)	2	2	2	1	2	2	2	2	15	93.8
Loyo et al, 2013, quantitative (28)	2	2	2	2	2	2	1	2	15	93.8
Yarnoff et al, 2019, quantitative (29)	2	2	2	2	2	1	1	2	14	87.5
Chen et al, 2018, quantitative (30)	2	2	2	2	2	1	1	2	14	87.5
Brittin et al, 2015, quantitative (31)	2	2	2	2	2	2	2	2	16	100.0
Apostolopoulos et al, 2018, quantitative (32)	2	2	2	2	2	1	2	2	15	93.8
Milstein et al, 2007, quantitative (33)	2	2	2	1	2	1	0	2	12	75.0
Jones et al, 2006, quantitative (34)	2	2	2	2	2	1	1	2	14	87.5
Ansah et al, 2019, quantitative (35)	2	2	2	2	2	1	2	2	15	93.8
Cruz et al, 2019, quantitative (36)	2	2	2	2	2	2	1	2	15	93.8
Homer et al, 2004, quantitative (37)	2	2	2	2	2	2	1	2	15	93.8
Diaz et al, 2015, quantitative (38)	2	2	2	2	2	2	2	2	16	100.0
Mishra et al, 2018, quantitative (39)	2	2	2	2	2	1	1	2	14	87.5
Homer et al, 2007, quantitative (40)	2	2	2	2	2	1	1	2	14	87.5
Diaz et al, 2015, quantitative (41)	2	2	2	2	2	1	1	2	14	87.5

Abbreviation: —, not applicable.

a Columns indicate score for meeting each of the following 8 criteria: column 1, presenting a clear objective; column 2, presenting clear scenarios and interventions; column 3, presenting clear outcomes variables by graphs, charts, or tables; column 4, describing the development of a system dynamics model framework or presenting a detailed model framework; column 5, presenting and explaining model parameters; column 6, improving the quality of data by using stakeholders’ engagement, surveys, interviews, and databases; column 7, validating models; and column 8, presenting a clear result. Because of the differences in evaluation indicators involved in qualitative and quantitative models, we used only 5 of our 8 quality criteria (criteria 1, 4, 5, 6, and 8) to assess the quality of qualitative research. We used all 8 criteria to assess the quality of quantitative research. The score for meeting each criterion ranged from 0 to 2 (0 = not mentioned, 1 = mentioned, and 2 = fully described).

b Qualitative research is a conceptual model for analyzing the dynamic complexity between variables in the system; in quantitative research, the quantitative relationships, various parameters, and equations in the system are determined and simulated for prediction.

c Qualitative studies have a top score of 10 and quantitative studies have a top score of 16. The higher the score, the higher the overall quality of the study.

d Percentage = the study scores divided by the total score for the category of study (10 for qualitative and 16 for quantitative) and multiplied by 100.

## Results

### Literature search

In our search for studies to include in our systematic review, we noted that most of the articles about system dynamics models of chronic disease prevention were from the US (n = 25). Other countries, including Singapore, Japan, Australia, Canada, Colombia, the Caribbean Community, and India, also carried out studies in this area (Table 1).

Generally, qualitative research uses a causal loop diagram to analyze the causality of variables in chronic disease prevention. A total of 7 studies used causal loop diagrams to analyze the causal relationship between diabetes, obesity, chronic kidney disease, and predictive measures (9–15). We developed a stock-flow diagram (Figure 1B) and a hybrid diagram with variable functions and parameters (Figure 1C) based on the causal loop diagram to predict the effect of various prevention measures on chronic diseases. Of the 27 studies that used quantitative models, only 3 studies established stock-flow diagrams (16–18). The hybrid diagram combined the advantages of the causal loop diagram and the stock-flow diagram, so it was used in the final simulation of the 27 quantitative studies.

### Upstream prevention

We examined the articles involving upstream prevention measures in detail. Among them, lifestyle approaches (balanced diet, moderate exercise, tobacco control, and mental health) were the most simulated prevention measures (n = 15, 44.1%) (9–12,19–29). Through simulation, the researchers found that lifestyle modification can reduce the incidence of chronic diseases, premature death, and medical costs among people with chronic disease, but the effect of lifestyle simulation prevention measures was more powerful in the long term (19–21,23–28). For example, the tobacco control program in Communities Putting Prevention to Work could prevent 45 million premature deaths and save $750 million in medical costs (20). Four qualitative studies used a causal loop diagram to analyze the causal relationship between lifestyles and chronic diseases (9–12). Researchers found that chronic disease screening showed a shifting of the burden. That is, when we took fundamental interventions such as lifestyles, there was no immediate effect from time delays in achieving the effect. People would turn their attention to the treatment of chronic diseases because treatment could quickly reduce mortality and complications.

Social factors such as employment, socioeconomic status, and community cohesion (n = 8, 23.5%) are also important aspects that affect chronic diseases (12,13,24,25,27,30–32). When the employment rate increased, the prevalence of chronic diseases was reduced to varying degrees (31). In addition, Chen et al (30) suggested that work stress or limited personal time would lead to an unhealthy lifestyle and increase the prevalence of obesity. Two qualitative studies concluded that primary health care was important for preventing chronic diseases, and that health policies, care quality, and cost were important factors affecting patient participation in primary care (12,13). Four quantitative studies found that effective healthcare measures can reduce the risk of chronic diseases, but cannot reduce cost (24,25,27,32).

Obesity is one of the important drivers of cardiovascular disease (CVD) and diabetes (n = 8, 23.5%) (9,16,21,22,25,33–35). Guariguata et al (9) proposed establishing qualitative models of diabetes through in-depth interviews with stakeholders in the Caribbean Community to determine the impact of obesity on diabetes prevalence. Simulation of quantitative system dynamics models showed that when the incidence of obesity was reduced, the incidence of diabetes or cardiovascular events was significantly reduced (25,33). Researchers found that prediabetes patients were still likely to develop diabetes after a period of preventive management, a so-called “backup” phenomenon (21,34). When the obesity rate decreased, no backup phenomena occurred, and more medical expenses were saved (21,34). Fallan-Fini et al (22) calculated the energy intake of different populations through system dynamics models and found that people with obesity had excessive energy (ie, no energy gap), which indicated that obesity would worsen further. Two quantitative studies (16,35) found that when the prevalence of diabetes and hypertension decreased, the morbidity and mortality from cardiovascular events and the prevalence of diabetic nephropathy decreased.

### Downstream prevention

Downstream prevention consists of clinical treatment and care of people with chronic diseases (n = 19 articles [55.9%]) (9,10,13–15,17,18,23,24,26,27,29,31,33,34,36–39). Some studies found that clinical treatment and care of people with chronic diseases could significantly reduce mortality. For example, we can achieve this long-term effect, mortality reduction, by improving the proportion of disease control and the affordability of drugs (10). Several qualitative models showed that patients with chronic diseases received continuous care, which was reflected in the improvement of health care quality and availability (10,13–15). Some studies reported that continuous care and access to health care after illness were important measures to reduce the recurrence of chronic diseases, mortality, and cost of care (18,23,26,37,38). However, clinical treatment can rapidly reduce mortality from chronic diseases without reducing their prevalence because of increased patient survival time (17,29,33,34).

Homer and Hirsch (4) and Homer et al (40) established system dynamics models to analyze the difference between upstream prevention and downstream prevention (n = 2 articles [5.9%]). The model simulation showed that when upstream prevention (lifestyles, social factors, and obesity) increased, the incidence of chronic diseases decreased. When people see the effectiveness of upstream prevention, more resources will be devoted to it. However, when downstream prevention increased, the mortality caused by chronic diseases decreased significantly. Because many chronic diseases are not curable, the reduction of mortality leads to more and more patients with chronic diseases living longer, which leads to the need for more resources for downstream prevention. Similarly, we also need to consider the issue of medical costs. Although clinical treatment of chronic disease showed significant medical cost savings in the short term, it was not beneficial in the long term (41). 

### Literature quality evaluation

The quality of all reviewed articles met our criteria, so none was dropped. Of the 34 articles reviewed, we found 30 good quality studies (88.2%), 3 medium quality studies (8.8%) and 1 poor quality study (2.9%) (Table 2). Seven articles used the Prevention Impacts Simulation Model (PRISM) but did not describe it in detail (19–21,23,24,27,29). PRISM is a that is used to simulate the effects of cardiovascular events and other chronic disease prevention measures. Nine articles conducted stakeholder interviews and used public data to ensure the quality of their studies (18,19,24,25,27,31,36–38). In 8 studies, only stakeholder or focus group interviews were conducted to collect data (10,11,13–15,28,32,39). Eleven articles carried out only sensitivity analysis or model data calibration for model validation and did not conduct a structural test and a behavior pattern test (17,26,28–30,32,36,37,39–41).

## Discussion

Our review evaluated the potential impact of system dynamics models on the upstream prevention (lifestyle, social factors, obesity) and downstream prevention (clinical treatment and intervention) of chronic diseases. System dynamics models can help us find the different results we expect from upstream prevention and downstream prevention, providing insights into prevention. We found that most models were created in developed countries such as the US. Chronic diseases were a huge burden in low- and middle-income countries, which showed an important research gap (42,43). Through systematic review, we found that the upstream and downstream prevention of chronic diseases had their own advantages and disadvantages. First, because of time delays in observing a desired outcome, the investment in upstream prevention (eg, lifestyle intervention, weight control) does not have an immediate effect, so more investment is diverted to downstream prevention (ie, preventing complications). When people focused on downstream prevention, the complications and deaths from chronic diseases were significantly reduced. However, limited medical resources make it impossible to prevent complications in patients with chronic diseases, and the death toll may rebound. With the increase of medical resources for upstream prevention, the incidence of chronic diseases and mortality from complications will continue to decline (4). However, the upstream prevention of chronic diseases takes a long time to exhibit the desired effect. For example, studies have shown that the clinical treatment of diabetes can reduce its mortality but cannot reduce its incidence. The reduction of the obesity rate would be more beneficial to reduce the incidence of diabetes (34). Therefore, the upstream and downstream prevention of chronic diseases has advantages and disadvantages, and the actual situation should be comprehensively considered to implement interventions.

Chronic disease prevention is a dynamic and complex process. Its complexity and time delays prevented us from discovering the consequences of prevention measures, which often led to unexpected results (3). System dynamics models are effective tools to help us transform system thinking into reality. For example, the most typical hybrid diagram is PRISM, which is a system dynamics model of CVD in the US (https://prism-simulation.cdc.gov/app/cdc/prism/#/resources). It can help health care workers estimate the impact of various prevention measures on prevalence, morbidity, and mortality from CVD, and the cost-effectiveness of such measures. Seven of the studies that we selected used PRISM to assess the short- and long-term effects of prevention measures on CVD or other chronic diseases to prioritize prevention measures (19–21,23,24,27,29). When the symptoms of the problem are the focus, without considering the root causes (eg, downstream prevention does not reduce the prevalence of chronic diseases), prevention alone cannot solve the problem. Instead, it could lead to more serious problems. Therefore, we can use system dynamics models to simulate the short- and long-term results of chronic disease prevention measures and choose the best measure for chronic disease prevention.

The establishment of system dynamics models is an iterative process, which deepens the understanding of chronic disease prevention through continuous learning. It can also help us understand the potential causal relationship between hidden assumptions and variables to better prevent chronic diseases. System dynamics models can help us understand the impact of feedback and time delays on chronic disease prevention and provide us with additional insights compared with traditional methods such as regression models. System dynamics models are also widely used in other aspects of the medical field, such as the prediction of health personnel needs (44). Not only can these models help rid us of local thinking (focusing on the part, not the whole), short-sighted thinking (focusing on the present, not the future), and phenomenal thinking (focusing on the surface rather than the essence) (45), but they can also help us capture the dynamic interactions of all aspects of chronic disease prevention.

Our study had limitations. First, we searched only 4 databases: PubMed, Scopus, Embase, and Web of Science. Other databases were not searched, which may have led to literature omission. In the future, we need to search more databases to obtain more evidence to explore the application of system dynamics models in chronic disease prevention. Second, we only evaluated the upstream and downstream prevention of chronic diseases, which may lead to incomplete evaluation. We need to explore other aspects of chronic disease research to enrich the results. Finally, the replicability of the system dynamics models was poor. We can set the model online or provide more raw data to solve the problem (ie, researchers can set the model to be available online, including variable equations, for readers to download for simulation and prediction, rather than obtaining only the schematic diagram of the model). Although our study had some limitations, we thoroughly reviewed the application of system dynamics models in the prevention of chronic diseases. The models can identify the relationship between upstream prevention and downstream prevention, which can provide some insights into the prevention of chronic diseases.

Our results showed that downstream prevention can greatly reduce mortality from chronic disease complications but cannot reduce the prevalence of chronic diseases. Upstream prevention, especially primary prevention, can greatly reduce the prevalence of chronic diseases. However, because of time delays, upstream prevention needs more time to show effectiveness, resulting in the resources for upstream prevention being preempted by downstream prevention. Therefore, we need to be cautious about the allocation of resources for preventing and managing chronic diseases. System dynamics models can connect different stakeholders who prevent, control, and treat chronic disease; help to understand the relationship between complex disease prevention measures; and provide insights for policy makers in chronic disease prevention.
